# Uses of Oranges

**Published:** 1888-04

**Authors:** 


USES OF ORANGES.—Oranges are said to render gastric fevers milder, even when of typhoid form. Eaten daily, before breakfast, they are said to remove the craving for alcoholic drinks, in old topers.—*Hering.*

